# Stability and electronic properties of gallenene†

**DOI:** 10.1039/d1na00553g

**Published:** 2022-01-17

**Authors:** Alex Kutana, Tariq Altalhi, Qiyuan Ruan, Jun-Jie Zhang, Evgeni S. Penev, Boris I. Yakobson

**Affiliations:** Department of Materials Science and NanoEngineering, Rice University Houston Texas 77005 USA; Chemistry Department, Taif University Taif 21974 Saudi Arabia; Department of Chemistry, Rice University Houston Texas 77005 USA biy@rice.edu

## Abstract

Two-dimensional metals offer intriguing possibilities to explore the metallic and other related properties in systems with reduced dimensionality. Here, following recent experimental reports of synthesis of two-dimensional metallic gallium (gallenene) on insulating substrates, we conduct a computational search of gallenene structures using the Particle Swarm Optimization algorithm, and identify stable low energy structures. Our calculations of the critical temperature for conventional superconductivity yield values of ∼7 K for gallenene. We also emulate the presence of the substrate by introducing the external confining potential and test its effect on the structures with unstable phonons.

## Abstract 2

Two-dimensional metals offer intriguing possibilities to explore the metallic character and other related properties in systems with reduced dimensionality.

## Graphical abstract

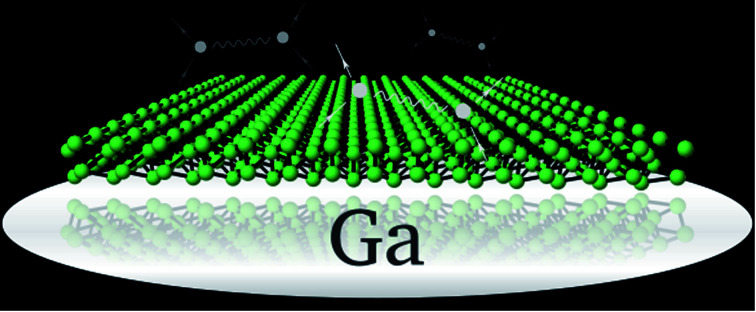

## 1 Introduction

Since their conception, two-dimensional (2D) materials have been of great fundamental and technological interest.^1–3^ Early 2D materials were envisaged as easily exfoliable, weakly coupled 2D layers with strong in-plane binding (*e.g.*, graphene, and transition metal dichalcogenides). More recently, however, there has been growing interest in exploring the possibility of two-dimensional forms where layering is not typical in the bulk form. One of the prominent examples of such a material is monoelemental boron, where none of the natural bulk phases are layered, but can exist in the 2D form.^4,5^ Further examples include silicene, germanene, stanene,^6^ and other non-van der Waals materials.^7^ Recently, another monoelemental 2D material composed of gallium – gallenene – has been isolated experimentally by solid-melt exfoliation on silica,^8^ grown epitaxially on the Si(100) and Si(111) substrates,^9,10^ or achieved by intercalation of epitaxial graphene on SiC.^11^ Located two rows down from boron in the periodic table, gallium in its 2D form is metallic, similar to borophene^12,13^ and other monoelemental 2D metals.^14^ Unlike borophene, however, which was originally grown on another metal (silver), gallenene was obtained on an insulating substrate, allowing exploration of the interesting properties of 2D metals with minimal interference from the substrate.

## 2 Results and discussion

Motivated by these advances, we conduct a computational exploration of the structural stability and properties of gallenene. Unlike previous studies,^8,15,16^ which focused on a limited number of gallenene structures, we aim at carrying out a comprehensive search employing the Particle Swarm Optimization (PSO) algorithm that allows the identification of low energy structures. In the structures with unstable phonon modes, we tested external potential confinement to emulate the stabilizing effect of the substrate, rather than applying an in-plane strain. We also evaluated the critical temperature for the superconducting transition from first-principles density functional theory (DFT) calculations in several gallenenes to study the effects of low dimensionality and nanoconfinement.^17,18^

**Fig. 1:**
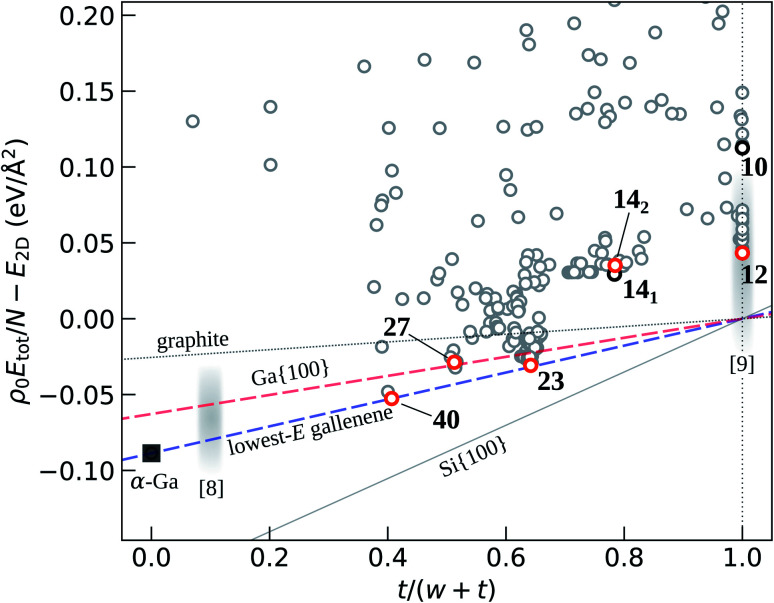
Total energy of gallenene structures per unit area per layer relative to that of the hypothetical most stable single-layer structure as a function of 1/*n* ≡ *t*/(*w* + *t*), the reciprocal of the number of layers, as defined in the text. This quantity is (within a constant *ρ*_0_) the energy per atom, or chemical potential of Ga at *T* = 0 K. Several low-energy structures studied here in more detail are labeled with red circles, whereas the experimental structures from ref. 8 and 9 are marked with gray fuzzy areas. Other high-energy structures are shown with gray circles. Structures are named based on the total Ga coverage, taking the Ga honeycomb as a reference, which is assigned a value of 10. The line connecting the lowest-energy gallenene structures and bulk α-Ga is drawn to show the trend, and compared with lines based on surface energies of graphite, Ga{100}, and Si{100}. From the line, the lowest energy of hypothetical single-layer gallenene is used as the reference *E*_2D_. The experimental point from ref. 8 is based on the reported thickness of ∼4 nm.

The CALYPSO code^19^ based on the PSO algorithm has been used extensively for predicting two-dimensional materials,^20–22^ and is adopted here to explore gallenene. Initially, random structures with certain symmetries are constructed with atomic coordinates generated by the crystallographic symmetry operations. To compare formation energies, structural optimization experiments are performed using the Quantum ESPRESSO code.^23^ Scalar relativistic ultrasoft pseudopotentials were used to represent ionic cores as provided by the pslibrary.^24^ The Perdew–Zunger local-density approximation (LDA) functional was employed. A plane-wave basis set with a kinetic energy cutoff of 60 Ry was used for the wavefunctions, and the Broyden–Fletcher–Goldfarb–Shanno (BFGS) algorithm was used for structural relaxations. Atomic positions were relaxed until forces on all atoms were less than 0.5 meV Å^−1^. In each generation, 70% of the structures are generated from the lowest energy structures in the previous generation using particle swarm optimization, while the rest of the structures are randomly generated. Here, we use a typical setting^19^ of 30 generations of gallenene structures, each with a population size of 20, yielding 600 structures in total. As shown in Fig. 1, gray circles correspond to all algorithmically-generated, optimized structures with relatively high energies. These points are shown for completeness and are not notated, since they are never referred to specifically but only as a collective set canvassed by CALYPSO. Among them, the structures with lowest energies, shown with red circles in Fig. 1, were investigated further.

The total energy *E*_tot_ of a layered material as a function of the number of layers *n* can be well approximated with the following expression:

1 *E*_tot_(*n*)/*A* = *nE*_2D_ + (*n* − 1)*E*_bind_,

where *E*_2D_ is the total energy of a single layer per unit area, *E*_bind_ is the binding energy between layers, and *A* is the area of the 2D cell. Here, a similar expression is adopted for bulk-like materials such as Ga. Substituting *n* − 1 = *w*/*t* and *nA* = *N*/*ρ*_0_, where *w* is the slab width (defined as the difference between the maximum and minimum of its atomic coordinates normal to layers), *t* is the thickness of a single layer, *N* is the total number of atoms in the system, and *ρ*_0_ is the assumed constant areal atom density in a single layer, and one obtains the following expression for the relative energy per unit area per layer:2
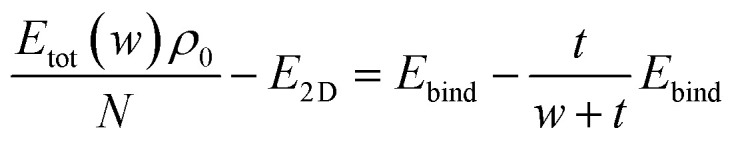


The first term on the left side of eqn (2) is essentially (within a constant *ρ*_0_) the energy per atom, or chemical potential of Ga at *T* = 0 K. For Ga, the layer thickness *t* was set to 4.4 Å, equal to the LDA *c* lattice constant of bulk α-Ga, giving *ρ*_0_ = 0.24 Å^−2^. For a non-layered material such as gallium, *E*_2D_ is the energy of a hypothetical atomically-thin structure obtained by extrapolating eqn (2) to *n* = 1. To estimate the “degree of 2D-ness” of gallenene, as shown in Fig. 1, we plot the left part of eqn (2) for the 2D Ga structures as a function of 1/*n* ≡ *t*/(*w* + *t*) and compare with both 2D-like and bulk-like prototypical materials such as graphite^25^ and silicon.^26^ We also plot the line based on the theoretical surface energy^26^ of the lowest-energy Ga surface, Ga{100}. The line connecting the lowest-energy gallenene structures and bulk α-Ga is also drawn, with its slope yielding the effective binding energy. Note that the interlayer binding energy *E*_bind_, *i.e.*, the energy of cohesion between the two layers, and the conventionally defined surface energy *γ* are related as *E*_bind_ ≃ 2*γ*. The experimental point is shown for comparison, based on the reported^8^ thickness of ∼4 nm for exfoliated gallenene.

One can see that in gallenene the energy trend with the thickness is quite close to one that would be obtained by cleavage at the lowest-energy surface, and actually shows a higher slope, *i.e.*, atomically thin Ga displays a bulk-like behavior. The effective binding energy obtained from the slope is 89 meV Å^−2^; this value should be compared with the binding energies of 26 meV Å^−2^ for graphite,^25^ 63 meV Å^−2^ for Ga{100}, and 176 meV Å^−2^ for the (2 × 1)-reconstructed Si{100}.^26^ The large slope may reflect the tendency of freestanding gallenene sheets to assume the bulk form. On the other hand, the low surface energy of bulk Ga may facilitate exfoliation, although the substrate and/or other kinds of confinement may play an important role in obtaining atomically thin layers.

Here the 2D structures are named based on the total Ga coverage, *i.e.*, the total number of atoms per area, taking the Ga honeycomb as a reference, which is assigned a value of 10. The energies of the previously considered^8^ gallenene structures, honeycomb *a*_100_ and rectangular *b*_010_, here designated as structures 10 and 14_1_, respectively, shown in Fig. 1, are seen to be rather high. Allowing out of plane displacements in the honeycomb *a*_100_ structure leads to a triangular-like structure with the 6-fold coordination, with an energy similar to that of 14_1_, designated here as 14_2_. Other low-energy structures are found, *e.g.*, 12 and 23, as shown in the ESI.†

We have performed the electron–phonon coupling and evaluated critical temperatures *T*_c_ for phonon-mediated superconductivity in several freestanding gallenene structures, as given by the McMillan equation.^27^ Phonon dispersions and electron–phonon coupling coefficients are calculated with the Quantum ESPRESSO code.^23^ The parameters and settings used are given in the ESI.† New structures 14_2_, 40, 27, and the honeycomb structure 10, are explored. All of the structures are metallic, as evidenced by their band structures and electron density of states shown in Fig. S2 and S3.† In addition, structure 14_1_ has a crossing of two bands near the Fermi level indicating the presence of a nodal line, as shown in the inset of Fig. S2b.† The Dirac-like band crossings at *J*_1_ and *J*_2_ in structure 14_2_ shown in Fig. S2c† are further below or above the Fermi level and are thus of less interest. The evaluation of the critical temperature is based on the microscopic theory of Bardeen, Cooper, and Schrieffer (BCS),^28^ with the rigorous treatment of electron–phonon interactions introduced by Migdal^29^ and Eliashberg.^30^ Phonon frequencies and electron–phonon coupling coefficients were calculated using the density-functional perturbation theory. The settings used in these calculations are given in the ESI.† The *T*_c_ values were obtained from the analytical approximation given by the McMillan equation,^27^ further modified by Allen and Dynes:^31^3
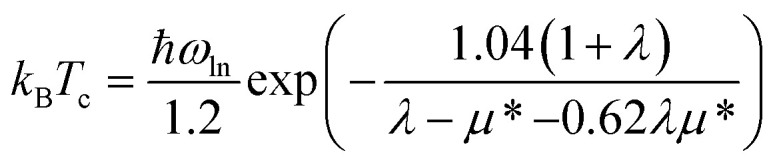


**Fig. 2:**
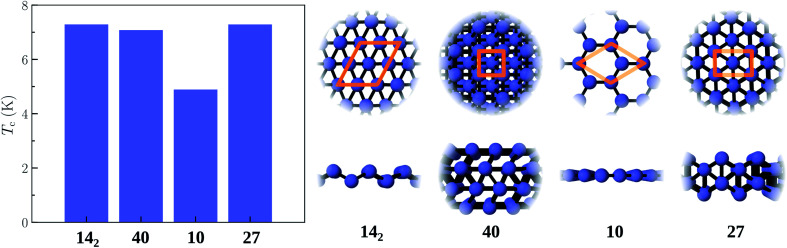
Calculated critical temperature *T*_c_ for phonon-mediated superconductivity in several gallenene structures. Top and side views of the corresponding gallenene structures (as labeled) are shown on the right, with unit cells indicated as well.

The prefactor *ω*_ln_ is the logarithmically averaged phonon frequency and the effective electron–electron repulsion is treated as an empirical parameter with the value μ* = 0.1.^32,33^ The obtained values of the *T*_c_ are in the 4–8 K range, as shown in Fig. 2, where the corresponding structures are also displayed. Similar values of the critical temperature are a result of similarity of phonon frequencies and electron–phonon coupling coefficients in different gallenene structures. A recent relevant paper^34^ brought to our attention after the submission of the present work has estimated the *T*_c_ to be 7–10 K for the structures taken from ref. 8 (unstable 10 and metastable 14_1_ in our notations, Fig. 1).

**Fig. 3:**
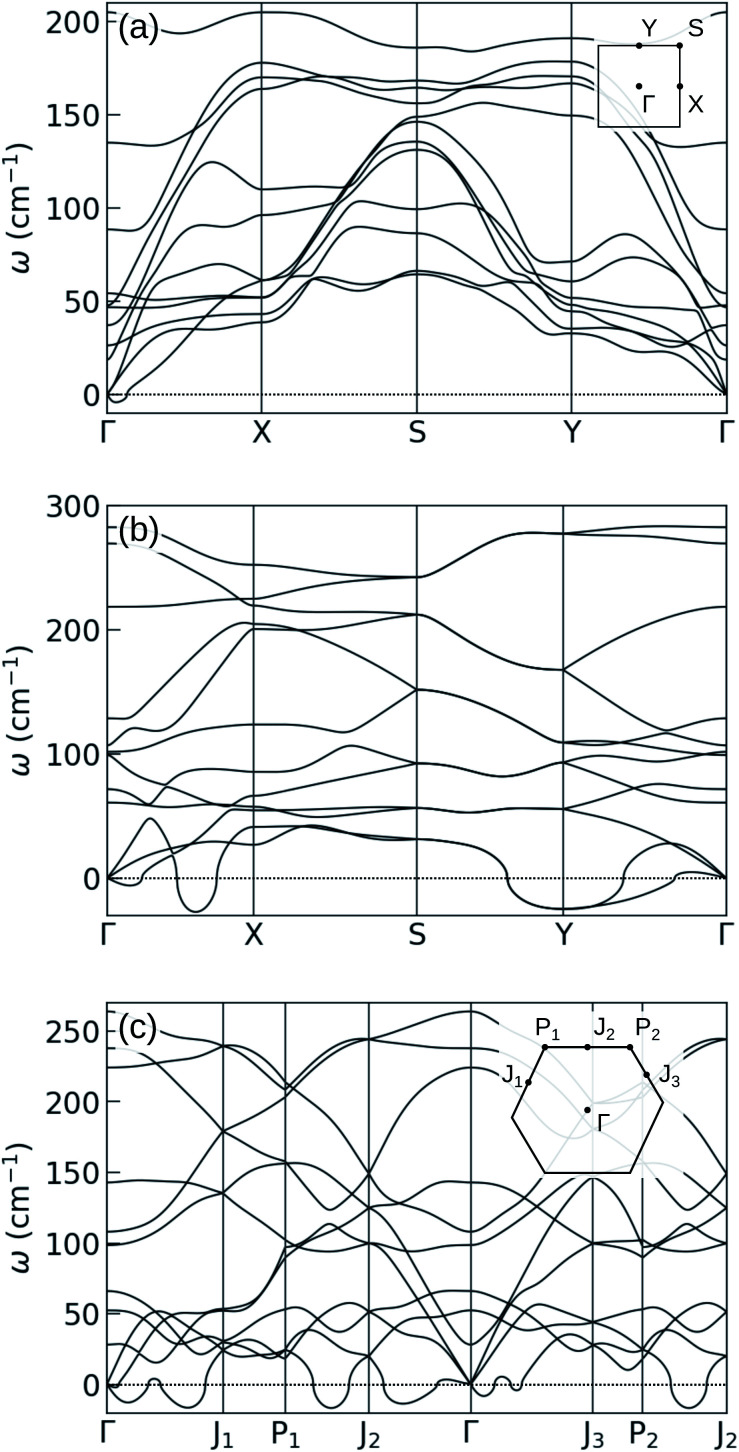
Phonon spectra of gallenene structures obtained from the search: (a) 40, (b) 14_1_, and (c) 14_2_. The corresponding Brillouin zones are indicated as well. For 2-layer structures 14_1_ and 14_2_, imaginary frequencies appear, indicating dynamic instabilities. These structures were considered to demonstrate the stabilizing effects of the substrate, as mimicked by the confining potential. Note that for 4-layer structure 40, the phonons are already stable without external confinement.

The phonon spectra of structures 40, 14_1_, and 14_2_ are shown in Fig. 3. The 4-layer structure 40 is stable, whereas structures 14_1_ and 14_2_ show phonon instabilities. Dynamic instabilities are common in freestanding atomically-thin 2D materials, manifesting as imaginary frequencies in the phonon dispersions obtained with DFT. Using supercells and applying small in-plane strains are some of the useful approaches for stabilizing the phonons in such cases. However, larger cell use and extra optimization are time consuming, while strains on the cell cannot imitate the stabilization effects from the substrate, as these strains are applied in the plane of the 2D materials (*xy*), but the role of the substrate is much like a confinement in the *z* direction. These confinement effects from the substrate can however be mimicked by applying an external potential.

The full interaction potential between the 2D adsorbate and the substrate is an unknown function of all the atomic coordinates. In the harmonic approximation however, one only needs to consider the second order term of the interaction. Therefore, an external potential of the parabolic form can be used to represent this interaction in phonon calculations. With the potential, the nuclear equations of motion are modified as follows.

In the adiabatic (Born–Oppenheimer) approximation and classical limit, the lattice-dynamical properties are determined from the harmonic phonon frequency *ω* which is the solution of the following secular equation:4
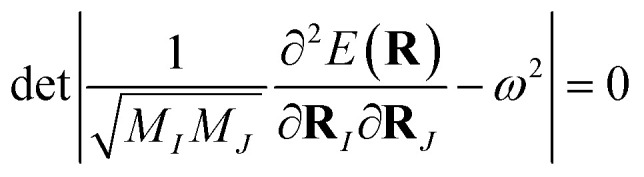
where **R**_*I*_ is the coordinate of the *I*th nucleus, *M*_*I*_ is its mass, **R** ≡ {**R**_*I*_} is the set of all the nuclear coordinates, and *E*(**R**) is the adiabatic potential energy surface. For monoelemental compounds such as gallenene *M*_1_ = *M*_2_ = … = *M*. After applying the external potential, the total energy *E*_tot_ becomes

5 *E*_tot_(**R**) = *E*(**R**) + *E*_ext_(**R**)

where, *E*(**R**) is the energy of the system without the external potential and *E*_ext_(**R**) is the energy of the external force field. A quadratic confining potential of the following form is applied:6
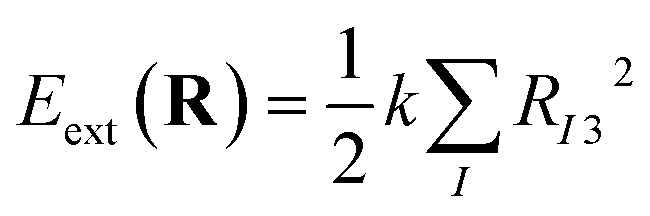
where, *k* is the force constant and *R*_*I*3_ is the coordinate in the *z*-direction of the *I*th nucleus. The corresponding force on the *I*th nucleus is given by7
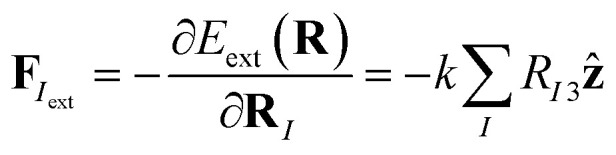
and its force constant *k* is added to the appropriate elements of the energy Hessian matrix. Based on the above equations, energy, force, and phonon subroutines of the Quantum ESPRESSO code were modified to implement the quadratic external potential, by adding the terms in eqn (6) and (7), and force constant *k* to the corresponding variables in the code.

In this work, a spring constant *k* = 3.7 kg s^−2^ was used. This value corresponds to a harmonic oscillation frequency of 30 cm^−1^ for the gallium atom, which is also the frequency shift of the ZA branch at the *Γ* point. The strength of this stabilizing potential should not exceed the interaction strength between gallenene and real substrates. Our DFT calculations show that this condition should hold for most substrates. For example, growth of gallenene on the Si(111) substrate has been reported recently.^9^ We carried out calculations for gallenene on the Si(100) substrate and obtained the second order interaction term *k* ∼ 56 kg s^−2^, which is ∼15 times larger than the value used to stabilize the gallenenes. The interaction curve between gallenene and the Si(100) substrate obtained from DFT is shown in the ESI.†

**Fig. 4:**
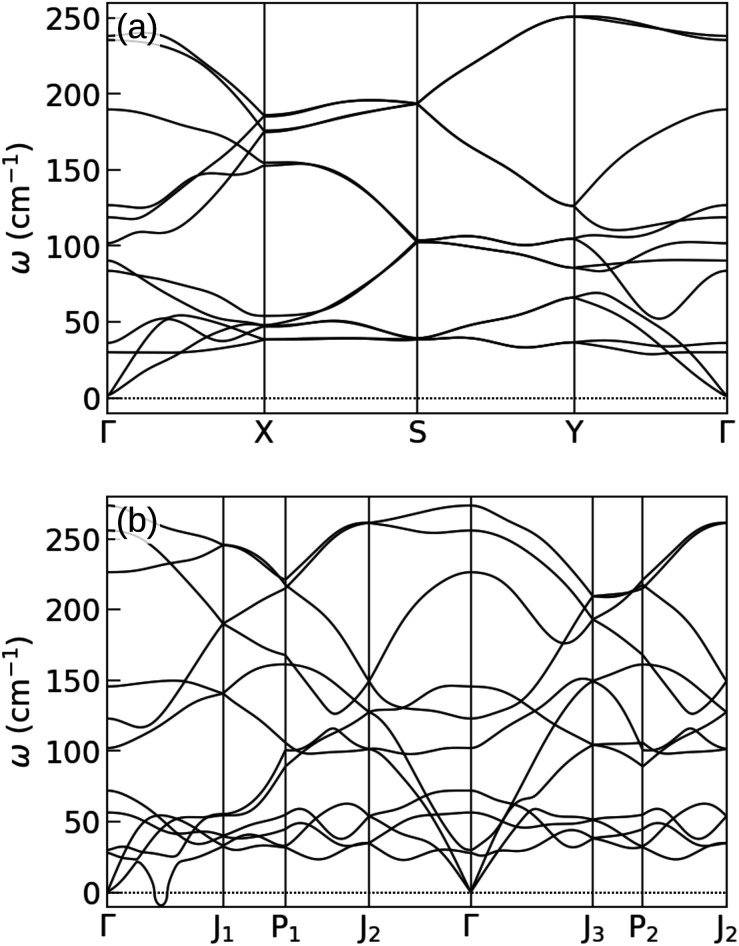
Phonon spectra of 2-layer gallenenes in external confinement: (a) 14_1_ and (b) 14_2_ structures which are also shown in Fig. 3b and c, respectively. Both structures flatten slightly after being put into the external potential. During optimization, in-plane lattice constants, angles, and atom positions are all relaxed. Notice that the ZA branches are all shifted to 30 cm^−1^. In the second structure, imaginary frequencies for in-plane phonon modes remain.

The obtained phonon spectra of structures 14_1_ and 14_2_ with the external confinement are shown in Fig. 4a and b. In the former structure, after re-optimization in the external potential, the imaginary frequencies are removed. The latter structure shows instability even in confinement, with imaginary frequencies existing between *Γ* and *J*_1_ points. The atomic motions in these unstable modes are in fact in-plane, whereas the forces from the external potential act in the *z* direction and thus can only stabilize the out-of-plane modes. This is the case for structure 14_1_, where the unstable modes along the *Γ*–*X* line are out-of-plane, and are stabilized by the external potential. The visualization of the unstable vibrational modes is provided in the ESI.†

## 3 Conclusions

In summary, we explored feasibility of two-dimensional gallium (gallenene) by performing a computational search of various two-dimensional polymorphs using the Particle Swarm Optimization algorithm. The stability trend of the structures found in the search points at the bulk-like behavior in few-layer gallium. An overall important conclusion seems to be that gallenene is unlikely to maintain the 2D-form, stable only at a rather thick “4-layer” form (structure 40) and gravitates toward thin films of bulk Ga. Only in the presence of the substrate can some thinner polymorphs be stabilized. This suggests that chemical vapor deposition or molecular beam epitaxy could possibly produce stable gallenene on substrates. Calculations of the critical temperature for conventional superconductivity have yielded values of ∼7 K in gallenene. Finally, external potential imitating the confining effect of the substrate has been applied to the structures that displayed unstable phonon modes. The potential is shown to be instrumental in stabilizing phonon modes with out of plane atomic displacements.

## Conflicts of interest

There are no conflicts to declare.

## Supplementary Material

NA-004-D1NA00553G-s001
