# Supplementary material for: Stability and electronic properties of gallenene
Source: Nanoscale Adv. 2022 Jan 17;4(5):1408–13. doi: 10.1039/d1na00553g (PMC9419834; doi:10.1039/d1na00553g)
Supplement: NA-004-D1NA00553G-s001 [file NA-004-D1NA00553G-s001.pdf]

Electronic Supplementary Material (ESI) for Nanoscale Advances.
This journal is © The Royal Society of Chemistry 2022

Supplementary information: Stability and electronic properties of gallenene

Alex Kutana,[a] Tariq Altalhi,[b] Qiyuan Ruan,[a] Jun-Jie Zhang,[a] Evgeni S. Penev,[a] and Boris I. Yakobson*,[b,c]

a Department of Materials Science and NanoEngineering, Rice University, Houston, Texas 77005, United States

b Chemistry Department, Taif University, Taif 21974, Saudi Arabia

c Department of Chemistry, Rice University, Houston, Texas 77005, United States

E-mail: biy@rice.edu

Interaction potential for gallenene on unreconstructed Si(111) substrate

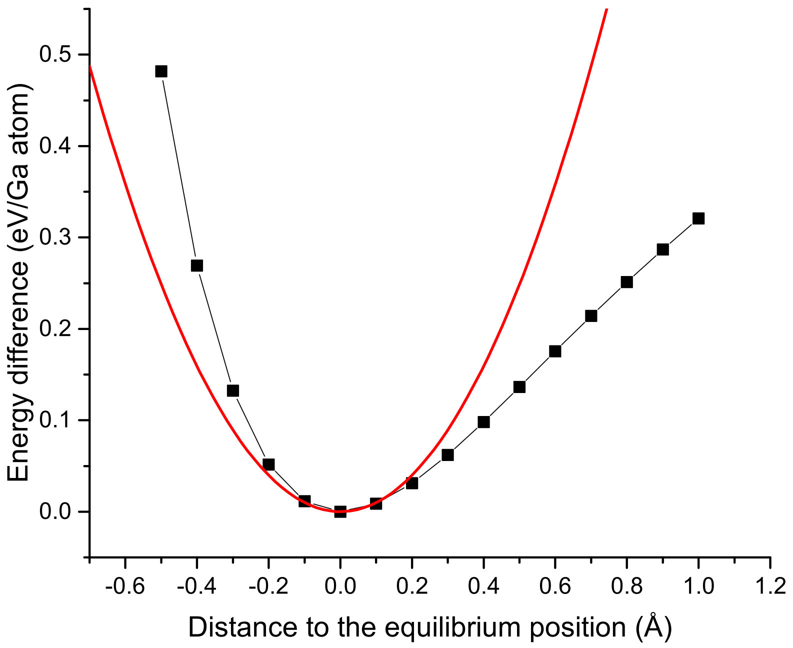

Figure S1: Interaction potential for gallenene on unreconstructed Si(111) substrate calculated with DFT.

Parameters for T_c calculations in gallenene

structure	k-mesh	q-mesh	$\omega_{\ln}$ (K)	λ	T_c (K)
$\mathbf{14_2}$	28×20	28×20	63.4	1.51	7.3
**40**	32×32	16×16	91.5	1.07	7.1
**10**	21×21	7×7	82.3	0.91	4.9
**27**	24×16	12×8	86.6	1.14	7.3

Band structure of gallenene

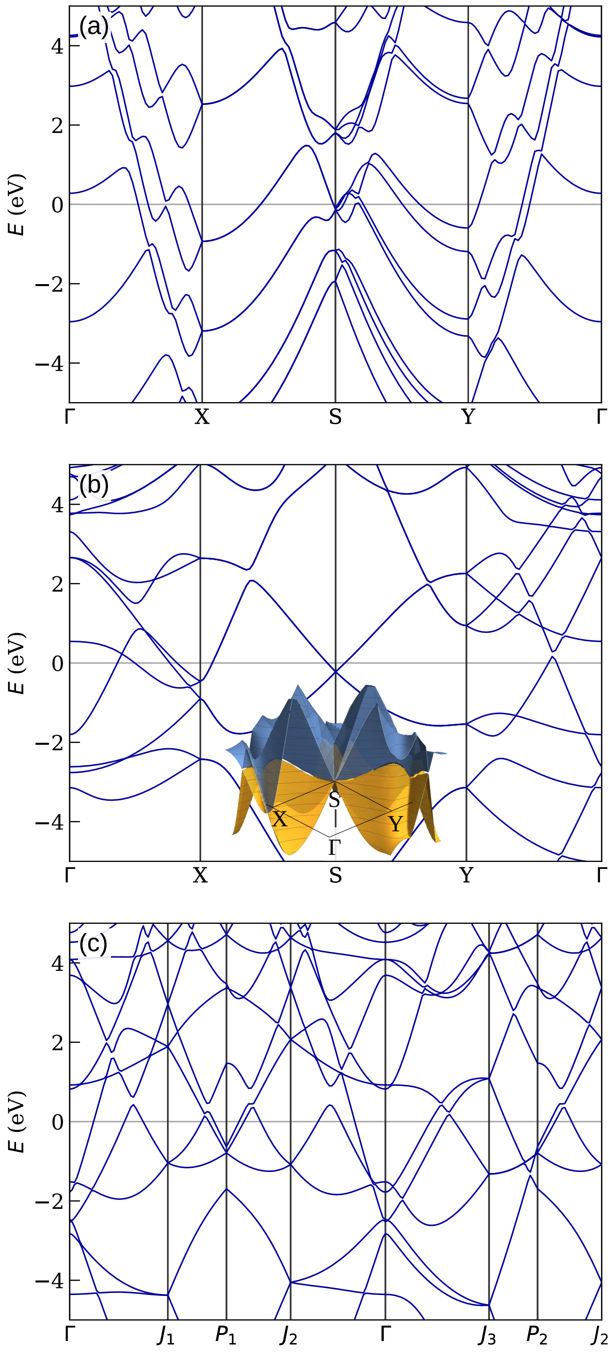

Figure S2: Electronic band structures of gallenene structures (a) **40**, (b) **14$_1$**, and (c) **14$_2$**. The inset in (b) shows the two bands that cross at the S point (rendered in the energy window $E_F \pm 2$ eV and only over 3/4 of the Brillouin zone for clarity), indicating presence of a nodal line near the Fermi level.

Electronic density of states

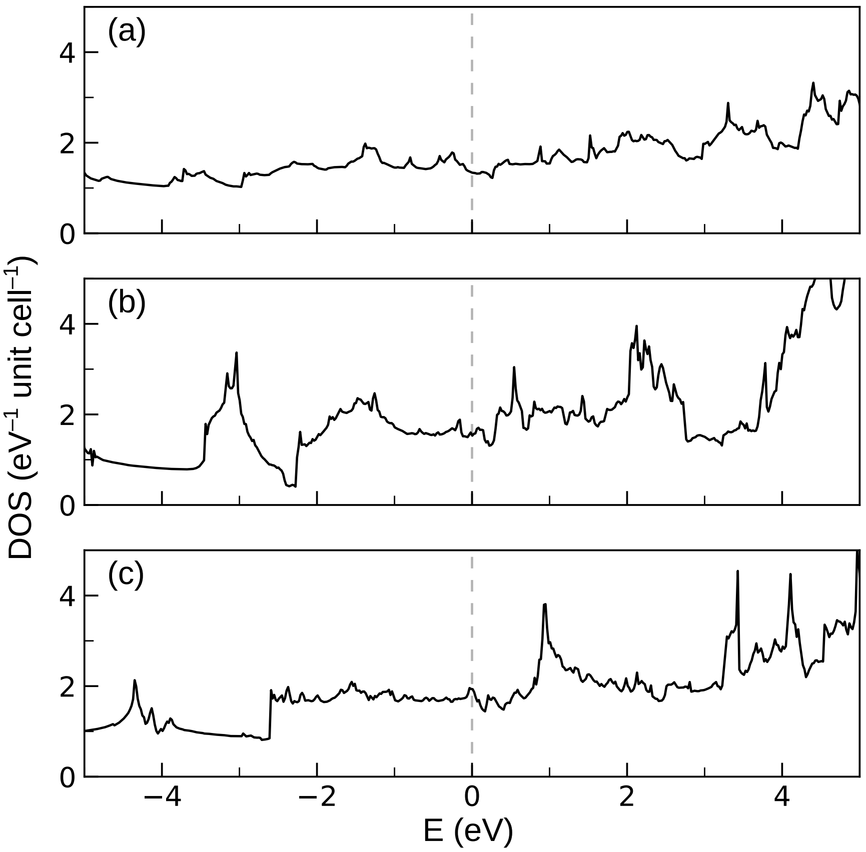

Figure S3: Electronic densities of states of gallenene structures (a) **40**, (b) $\mathbf{14_1}$, and (c) $\mathbf{14_2}$.

.

Gallenene structures 12 and 23

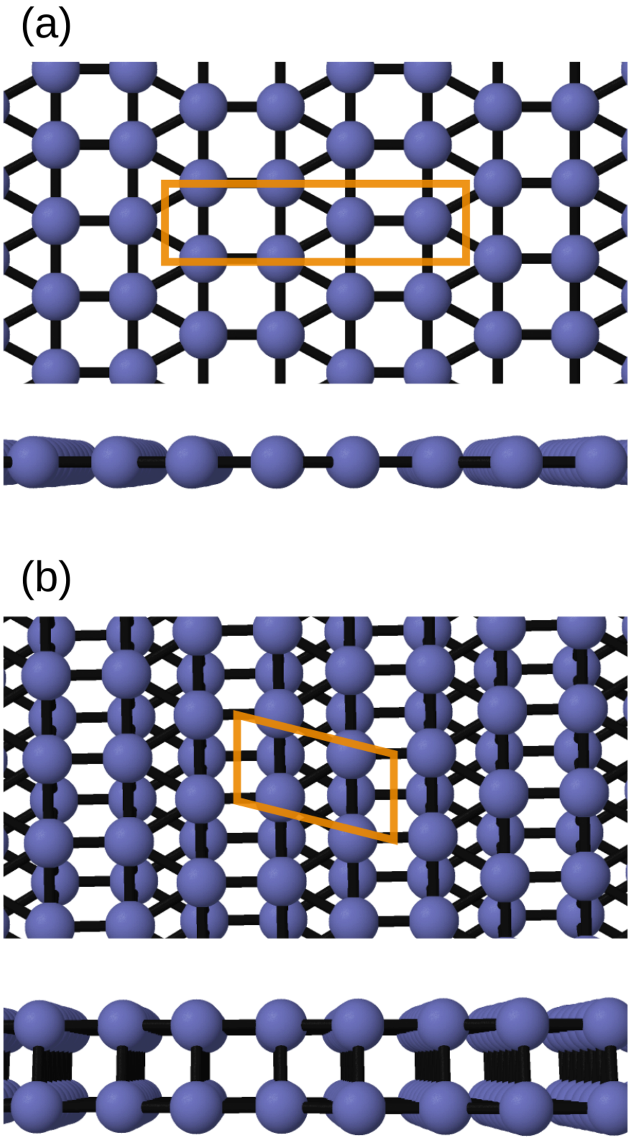

Figure S4: Gallenene structures (a) **12** and (b) **23**.

Visualizations of unstable out-of-plane and in-plane phonon modes

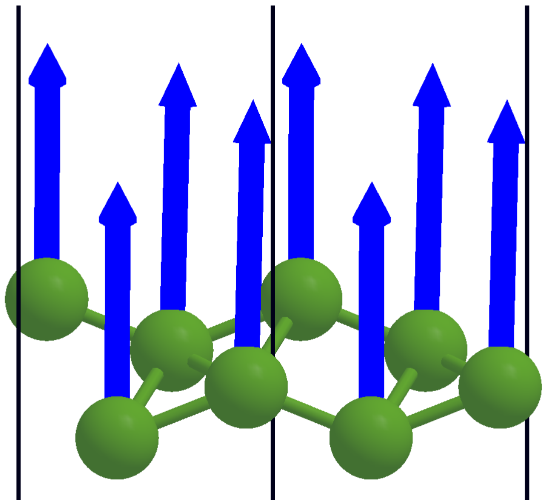

Figure S5: Visualization of the unstable out-of-plane phonon mode along Γ-X direction in Figure 3b of the main text.

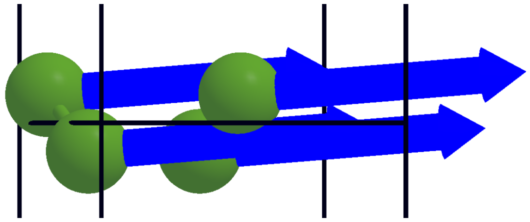

Figure S6: Visualization of the unstable in-plane phonon mode along Γ-J_1 direction in Figure 4b of the main text.
